# Local Experiences in Community Health: Examples from Canada, Brazil and Mali, and an Experiment in France

**DOI:** 10.3934/publichealth.2015.3.537

**Published:** 2015-08-21

**Authors:** Sébastien Fleuret

**Affiliations:** 1 CNRS, UMR ESO, University of Angers, Maison de la recherche G. Tillon, 5 bis bd de Lavoisier 49000 Angers, France

**Keywords:** community, health, places, international comparison, geography

## Abstract

This paper presents the findings of international research with an original approach anchored in health geography, which illustrates the importance of place as a dimension in community health. The aim of the research is to identify the success factors in the processes used to build community health initiatives at the local level. The study is based on interviews encoded and analysed using the framework of the grounded theory. Three main themes—the place, the community and healthcare supply—and two cross-cutting issues referring to 18 explanatory dimensions are identified. These findings are then put to the test in France through an action research approach. Overall, the work suggest avenues to enable the transferability of successful elements of community health initiatives.

## 1. Introduction

After a period of relative oblivion since Alma Ata (1978), we now observe a re-emergence of community health in connection with a growing concern for primary care, and for the structuration of health determinations at the local scale [1], [2]. Non-medical determinants of health are receiving greater consideration [3]. This has given rise to a paradigm shift: the health (and no longer just healthcare) available to populations is now the subject of a transversal, cross-sectoral and holistic approach [4]. This evolution is also spatialized: the holistic approach to health only exists at the local scale and is limited to basic care and services, whereas the more specialised levels of care and services remain centralized and in the hands of the hospital [5].

In relation with this evolution, we observe growing attention being paid to basic services, to non-specialised actions and to health education, along with its promotion outside the biomedical field [6]. Health questions are no longer the exclusive province of medical experts. These trends are occurring in the context of a general withdrawal of the state from provision of public services, and a reduction of the funds allocated to health care structures. This has led to transfers of responsibility from the public sphere to the private sector and to the community sector (also called third sector) [7]. However, these developments unfold in different ways in different places, as can be illustrated with reference to community health initiatives: these initiatives are the core of the field studies presented in this paper.

Local community health initiatives are numerous and deserve attention because the developments mentioned above set the context for of using these initiatives as models which can be developed, transferred or generalized in the face of pressure on public health systems—in northern countries as well as southern countries [8].

Although the transferability of community development initiatives has been studied, it remains a largely fuzzy concept, and some authors underline a lack of evidence and rationale [9]. However, the literature points out that successful initiatives result from both the implementation process itself and the context in which it is developed [10]. The notion of success in local health initiatives is likely a combination of “grassroots” and funding factors (needs, available resources, social, economic and geographic context etc.) and a utilitarian logic. These must be correlated, otherwise many health programs turn out to be failures because the services they provide, and which promoters deemed to be adapted and sufficient, are hardly if at all used by the population [11].

The position adopted in this article is geographical and consists in studying contextual factors rooted in territories. Its focus is on the range of programs and actions existing at local scale - specific factors that may enable success in local community health initiatives but also present challenges for transferability. To meet this objective, three questions must be addressed: What factors impel the appearance of local community health supply in a specific place? How does this emergence settle into a long-term structure? And why is it so rarely possible to generalise and reproduce the results of these examples elsewhere?

These questions are not asked outside any operational framework, they rather are based on fieldwork observations. This work started in Quebec, where the delivery of social and primary care services is territorialized at the local scale. Although two reforms (in 2005 and 2014) challenge this organization, the community Clinic Pointe-Saint-Charles (CPSC), established in 1968 as a model for setting up a Quebec network of local community services centres, remains in operation. The CSPC has maintained its community basis while the health care system in Quebec has been modified by successive reforms leading to more integration and merging of local structures, taking other health care services away from the local and community scale. Besides economic considerations, these reforms lean partially on reports that the transposition of the initial community model did not completely satisfy all needs.

From this initial observation, opening the view towards other countries allows us to add a comparative dimension, and to distinguish local contextual specificities from general patterns. This paper presents the findings of research conducted in three international fields with the aim of identifying the factors which made it possible to develop a community health supply at these particular locations. Findings from this work were then put to the test in France, in the Town of Angers.

The fields of study are presented in the first section, which includes a justification of site selection. The second section describes the method used. In the third section, the results achieved from international field work are discussed. The fourth and final section deals with the way these findings were tested in 6 neighbourhoods in Angers, using an action research approach.

## 2. The fields of study

### 2.1. Choice of Fields

The fields were chosen according to criteria aimed at completing a comparative study based on both common and discriminating elements. These elements made it possible to highlight the subtle differences between commonalities that can be reproduced whatever the context and specificities stemming from geographical context.

The first criterion was to consider the services based on a holistic approach to the person. This implies that a person's entire life situation needs to be assessed and, above all, calls for services to be provided locally so as to be able to act on the non-medical health determinants. A partnership involving a maximum number of actors is required to make this possible [11], [12].

As second criterion, it seemed essential that the fields chosen should be places where experiments with a participatory aspect had been carried out and positively evaluated. This is because health promotion can be understood as *“the result of an effective and active participation of the community in the setting of priorities, in the decision making and in developing planning strategies to achieve a better level of health”* [13].

The third criterion was geographical: the case studies were all territorialised, in the sense that each territory was well-defined and clearly delimited [14]. Fourth, all these cases could be considered exemplary, because they were or had been identified as examples which could potentially be used as models. But the attempts to implement these models in neighbouring territories had not yielded the same results as in their original sites. Finally, the choice of fields was guided by an essentially operational requirement. It was imperative to be able to identify and meet freely with the health sector, territorial and civil society actors involved in each case.

Thus, we began by choosing and working in three fields (in Montreal- Canada-, Bamako-Mali- and Camaçari-Brazil-). Then a French field was selected to test the relevance and the operational effectiveness of the results obtained in these three cases. The idea was to test the results in a country which is absolutely not organised in a way that supports the formation of community structures offering frontline care. Although a few experiments exist, such as municipal or mutualist health centres, community health in France is still at a very early stage. In the end, the “collectifs santé” (health collectives) of the municipality of Angers were chosen as case studies. These initiatives have been developed in the frame of the CUCS (Urban Social Cohesion Contract), which is the local part of French urban policy.

### 2.2. Presenting the Three International Fields

#### 2.2.1. The Community Clinic of Pointe-Saint-Charles

The Pointe Saint Charles neighbourhood is located south west of Montreal, Quebec on the banks of the river Saint Laurent. It is one of the oldest neighbourhoods in the town and its history is closely linked to the industrial development of Montreal. The deindustrialisation process, which began in the 1960s, had a major impact on the living conditions, which deteriorated as unemployment rose and businesses and services closed down.

The Community Clinic of Pointe-Saint-Charles (CPSC) was launched in 1968 on the initiative of some young medical students from McGill University, along with some inhabitants of the neighbourhood faced with the shortcomings of the existing system. In the 1960s “*there are few doctors, none of them work full-time and only one actually lives in the neighbourhood. There is 1 doctor for 6,000 inhabitants whereas the Canadian average is 1 doctor for 857 inhabitants (…) There is only a limited access to public medical services and most of the time families have to pay for the consultations. As there is no hospital in the neighbourhood, people have to travel to get healthcare. Quite an expedition for most of them as they don't have a car.”* [15]. When it was created, the CPSC sought to follow the model of the free clinics found in some deprived neighbourhoods in the USA [16]. These free clinics endeavoured both to offer services and to give citizens a role in the administration and the distribution of healthcare and related services, such as paramedical or social services. The CPSC went on to become a model itself in the Castonguay-Nepveu report (1967-72), which is the founding document of the modern Quebec health system. Amongst its many recommendations (106), the report suggested creating locally-based centers providing community services. The first ones would appear in 1974 (for a detailed history, see [17]–[19]).

When creating the CLSCs, the reformers adopted the health conceptions that prevailed within the community health clinics [20]. However, the CPSC has always refused to become a CLSC, in order to maintain its independence. This was a very committed stance, which led to many collective movements aiming to counter the different attempts to merge the clinic with the public network (in 1974, 1978, 1992 and recently in 2004^1^). These struggles allowed the clinic to keep its autonomous community structure status. The clinic continued to develop its services and currently has a staff of 125 permanent employees who provide the population with a large range of services. According to estimates made by the clinic administration, about 30–40% of the inhabitants of Pointe Saint Charles use these services.

#### 2.2.2. The Banconi Community Health Centre (Cescom)

Banconi, a working-class neighbourhood to the east of Bamako, Mali is the result of a rural exodus phenomenon, which over the years gave rise to informal housing at the foot of the cliffs of Kouboula. The configuration of the neighbourhood only began after 1987, with the development of a poorly-structured urbanisation. Until then, Banconi had no tarred roads, no street lighting, no private power supply, no water supply system and of course no sanitary equipment. Little has changed and Banconi is still a deprived and under-equipped neighbourhood.

A survey on primary healthcare and traditional medicine, led by the National Institute of Public Health (Mali), found that households spent less than 3–5% of their budget on health. This is linked to sky-high healthcare costs and excessive distance from the hospital [21]. From the highlighting of this widespread need stemmed the idea of creating an “Assaco”. (Community Health Association). The Association of Banconi was the first Assaco in Mali. It arose from the observation that there was a double problem of poverty and difficult access to healthcare. It was therefore in response to a need that primary health services were implemented, starting with a small hut with medicine, a doctor, a midwife and a security guard [22].

This association went on to create a Cescom*—“a private non-profit health centre, which is managed by a users association and is comprised of a medical clinic, a maternity clinic and a pharmaceutical warehouse. Manned by a team of healthcare professionals led by a doctor or a nurse, it provides the surrounding populations with primary healthcare”* [23]. On March 9^th^ 1989, the Cescom was created with no money, no means and no help from the State (which did not favour the initiative). However it did have the support of public health researchers. The absence of public authorities was compensated by the help of the French Development Agency. The building was financed by the Angers-Bamako partnership and by bequests. Today these arrangements suffice to cover the operating costs, especially as they are sometimes supplemented by funds from non-profits as well as by national programmes (e.g. for vaccinations). The Banconi Cescom has often served as example and to date 350 Assacos and Cescoms have been set up all over Mali, consistent with the idea of a territorial grid.

#### 2.2.3. The Camaçari Family Health Programme (PSF)

Camaçari is a town with 242,000 inhabitants in the State of Bahia, Brazil. This example is original because, after emerging from a dictatorship, Brazil had to quickly develop basic health services while taking into account territorial issues [24]. This led to sharing out the health responsibilities over three levels: the federal government, the states and the local municipalities. Primary healthcare is the prerogative of the municipalities.

From an epidemiological standpoint Brazil completes our range of profiles. Canada has completely undergone its epidemiological transition (ageing population, intensification of chronic diseases). Mali is still dealing with contagious diseases and a high rate of infant mortality. Brazil is situated between the two: infant mortality is decreasing thanks to a high level of vaccinations, and little by little contagious diseases are being brought under control as medicine becomes more easily available to the population.

The healthcare structure studied in Brazil is the Family Health Programme (Programa Saude da Familia-PSF). It is the local component of the Unified Health Service (Sistema Unico de Saude-SUS), which was created in 1988 upon the return of democracy. The SUS is based on 5 main principles which take form through the application of the PSF (Table 1).

**Table 1.** Unified Health Service and Family Health Programme in Brazil.

Principles of the Unified Health Service	Translation of these principles in the PSF at the local scale.
Universality	Responsibility to provide a service for each territory
Equity	Targeting of each territory's needs
Comprehensiveness	Articulation of education practices and health and healthcare promotion. The PSF is the gateway into the system
Social participation	The community is involved in the planning and the local health councils exercise an a posteriori monitoring and a retroactive budget control.
Decentralisation	The practices are reshaped from the primary healthcare

The originality of Brazil, compared with the two other fields of study, is that the implementation of local health services is not a case of starting from an example in order to generalise it. Rather, the PSF is a way of working, which is implemented at the municipal and infra-municipal levels. The principle is as follows: a PSF team is set up on each territory with at least one doctor, one nurse, two auxiliary nurses and six community health workers. These teams must pinpoint and plan promotional, preventive, diagnostic, treatment and rehabilitation actions for the population (about 1,000 families per team).

The same methodology is applied each time: first, a territorial diagnosis is carried out. This is the identification phase, which makes it possible to pinpoint the health problems, to plan actions and to foster future cooperation with the other actors in the field. In the second step, a transdisciplinary team is put into place to address promotion measures (directed at the general population), preventive measures concerning certain problems (aimed at the population at risk), and other curative actions. The family and its social space are the heart of the healthcare support. Community workers visit families on a monthly basis and carry out actions to promote health and prevention as well as following certain groups. The implementation of the PSF in Brazil has been taking place since 1998 and today over 60% of the country is covered.

## 3. The method of analysis

This research uses the grounded theory framework [25]. Its principle is the systematic generation of a theory from qualitative data, in an inductive manner, using predefined stages and constant back-and-forth between the collection and the analysis of data. The field work consists of conducting “enlightening interviews”, with the aim of gathering points of view on a subject as though lighting up a scene from all the different angles. In situations were the number of angles remains numerous, the goals of simplification and generalization can be achieved by creating not a single model, but *“a family of sub-models, each one of them summarizing as accurately as possible a series of examples close to each other. According to studied reality, the sought-after goal and the degree of accuracy wished, the sub-models will be more or less numerous.*” [26].

The interviews took place with healthcare staff (medical and non medical), administrators of structures providing health and social care services, elected representatives, supervisory representatives and community representatives (from associations and civil society). In accordance with the grounded theory, the interview grid was developed as information was collated, while keeping a fixed frame for comparison purposes. Each interview could also serve as an opportunity to identify a new interlocutor able to offer fresh insights. The interview frame was semi-structured. The number of interviews was determined by means of the information saturation principle (that is to say, when new interviews bring no new information and when there are no more grey areas).

The interviews were carried out in Montreal first, then Bamako, followed by Camaçari, before a return to Montreal to complete interviews. In all, 35 interviews were conducted, transcribed and coded, using NVivo software to make a structure emerge (Table 2). The coding procedure was carried out using the open coding method.

Initially, the interviews transcribed in the analysis software were given markers labelled “nodes” by NVivo (N = 883), which served as anchors. These nodes were then grouped in the software by similarity in order to form hierarchical nodes (N = 407). These hierachical nodes were grouped once again to make up explanatory dimensions (N = 18). A final grouping by proximity was made, leading to the identification of three main themes - the place, the community and healthcare supply - and two cross-cutting issues. This is the reading frame for the results. All these grouping phases were realised within the software, which keeps track the origin of each quote at each stages of the analysis. So it is possible, at the last step of the synthesis, to know which interviews contributed to each of the 3main themes and 2 cross cutting issues finally identified, and which exact terms were used.

**Table 2.** The four steps of the coding process in NVivo using the principle of the grounded theory.

Step 1	Step 2	Step 3	Step 4
Nodes	Hierarchical nodes	Explanatory dimensions	Main themes
**883**	**407**	**18**	**3+2**

### 3.1. Cross-comparison of results

The results here after are presented according to the 3 main themes and the 2 cross-cutting issues. For each a description of the explanatory dimensions contributing to their character is provided. Some explanations may be closely similar, which can be explained by the fact of overlap in the coding process—e.g. two hierarchical nodes may have contributed to two explanatory dimensions. To reduce overlap, where nodes appeared in more than once, the analysis focused on the context in which they were more important (estimated by the frequency of citations—quantitative appreciation—or by the vehemence of the discourses). Some overlaps were retained where they could be distinguished by nuances of meaning—these are signaled in the text by a footnote.

### 3.2. Building Places

Five dimensions help to characterize how important the place is in the implementation of local community health projects (Table 3)

**Table 3.** Dimensions related to places.

Dimensions
Place and scale effects (1)
Decompartmentalization (2)
Decentralization (3)
Size effects (4)
Horizontal set up (5)

The geographical location creates place and scale effects (1) which need to be taken into account, and can partly explain the difficulties faced when transferring an experiment from one place to another. For instance, in Camaçari an interviewee mentioned ties of *familiarity and complicity with the population.* These ties mean that *after it is easier to convince them to adopt healthy behaviours.* For example, it is important for the doctors and nurses to be residents of the territory. (2) Territorializing healthcare encourages comprehensive care of the individual as a whole—as opposed to emphasizing the medical aspects while neglecting other factors. Thus there is a need for decompartmentalization: health professionals have to collaborate with other actors in the place (e.g.. social workers, agents of the municipality). For instance, in Bamako it appears that “*curing” malaria is not simply a question of drug availability but also of sanitation, the treatment of water sources, the distribution of mosquito nets.*(3) So malaria is a problem requiring an integrated response from a number of actors on a territorial basis. An example of such a territory is Camaçari, where there is a local government project on the typology of the area according to health risks and other indicators, such as the number of social security recipients etc. (4). This implies that decisions are decentralised to some degree and that local initiatives are fostered. Thus, in Montreal *the Clinic has maintained its “responsibility to the population” and its status as a community organisation, therefore it is not a subcontractor for the health and social services centre^1^*. (5) Similarly, size effects are at work for the same reasons, which are the ability to act and the ability to make decisions locally. At the community clinic of Pointe-Saint-Charles, one person compared their structure to the CLSCs which make up the rest of the primary care network in Quebec: *There's the word community in CLSC but it has nothing to do with the idea of community. It's too large-scale: it's an institution. Here it's really small, you really can't compare (...) As far as I'm concerned, big and community just don't go together.*

### 3.3. Building Communities

In the field of health, policy makers and stakeholders seldom define ‘community’ geographically, rather they rely on its representatives to provide a community justification for the technical choices they make [27]. In the three fields of study the link between the healthcare supply and the population which makes up a community is described as essential. This is reflected in the 5 following dimensions (Table 4).

**Table 4.** Dimensions related to communities.

Dimensions
History, Identity (1)
Empowerment, appropriation, consideration for popular knowledges (2)
Social context, collective challenges to overcome (3)
Activism, mobilization (4)
Resistance (to privatization, gentrification, institutionalizing (5)

(1) The first dimension is the history of the places and their identity, which shape a common experience. *In order to make this [initiative] last, we managed to create a tradition* (Pointe-Saint-Charles, Montreal). (2) The second dimension is that of e*mpowerment,* of the appropriation of health tools by the population, which goes hand in hand with the recognition of popular knowledge. This is what leads to achieving a balance between public policy and the expectations of the community. Otherwise, the health centres are not frequented, as can be observed in Bamako for example.

The problem is that a building is erected then people are told that will be their health centre but the population hasn't even been involved. (3) The social context is another factor of community cohesion which can bring together the initiatives in the community, particularly when there are collective problems to overcome. Thus, for a doctor in Camaçari, there is no need to take people's blood pressure because I know about the pressure due to the prevalence of violence in the neighbourhood. So working on healthcare in the community means, in this case, above all dealing with this issue.

This ties in with the following dimension, which is that of activism and mobilisation (4). The community structures of the health initiatives are not ad hoc objects, they are the result of militant commitment: *a doctor who is not militant and committed cannot last. Commitment is a very important criterion when recruiting. You need activism and a certain degree of community involvement* (Bamako).

A particular form of mobilisation seems to strengthen community building and provide support to undertake actions in frontline healthcare. This is the posture of resistance which can take many forms (5): resistance to the privatisation of healthcare, to the gentrification of the neighbourhood which denatures the community, to institutionalisation...

*Gentrification mobilises people. They feel that they are losing the territory. The people who do up the condos don't need the local schools, they don't go to them. They don't need the CLSC or the clinic. So people here are afraid that they will no longer have the means to stay in their neighbourhood. Dog parks are created for Mrs Condo, who owns a dog, but there's a lack of parks for the neighbourhood children* (Montreal).

### 3.4. Building Healthcare

Although it is central, healthcare supply is not the first thing the people interviewed chose to highlight. This is likely due to the fact that all of them are convinced that health is something global and cross-sectional, and so they highlight the territory and the community as supports before mentioning health as an object of public policy.

There are 6 dimensions which contribute to defining community healthcare (Table 5).

**Table 5.** Dimensions related to healthcare.

Dimensions
Organization of professional practices (1)
Professional networks (2)
Human relations (recognition and mutual trust) (3)
Acknowledgement of the person as a whole (4)
Response to needs, quality of services (5)
Autonomy to manage the resources (6)

First of all, there are the ways in which professional practices are organised (1): collective organisation, the coordination of actors, especially when they are very dissimilar and belong to different, sometimes compartmentalised, institutions. In Camaçari for instance, the existence of a municipal coordinator whose role is to bring the actors from the healthcare sector to work together is put forward as an asset.

The second dimension, linked to the first one, is the flow of information, the “networking” (2). This concerns exchanges professionals between themselves but also with the population, which is particularly promoted in Montreal: *there is an exchange, the others also can teach us a thing or two. (Montreal)*

In these exchanges, it is more about human relations (mutual trust, recognition) than simply organisational arrangements (3). Knowledge of one another reinforces the cohesion of the actors in the health sector and improves the practice of healthcare. A patient-care provider proximity develops and makes it possible to approach health in a comprehensive manner, taking into account the person as a whole, contrary to a cold consultation leading to a strict biomedical diagnosis. *Human warmth: here people aren't numbers, they aren't customers, they are participants* (Montreal).

The following dimension consists of the participation of the population in local health decisions (e.g. management of a community health centre) (4), particularly through the idea of a right to a say. If the aim is for health to concern everyone (Ottawa Charter, OMS) then everyone must have the right to a say on the services provided. This can be within, for example, a participatory administrative board (Montreal, Camaçari).

The importance of good quality services (5) is underlined by all the people interviewed. In the three fields of study, initiatives such as health assessments of the territory were carried out in a more or less formal manner. In Camaçari, this process also concerns healthcare planning at a higher level. *The situation has been reversed: before the local level would relay requests which were subsequently integrated into the planning. Now a diagnosis of the problems is made by the planners, who then go down to the different territories in order to provide solutions to the observed problems.* (Camaçari)^2^

Finally, those interviewed evoke the need to dispose of resources (financing, equipment, services) and to be able to manage them locally with a certain amount of leeway and autonomy (6)^3^. *The financing by programmes conditions and also influences practices* (Montreal).

### 3.5. Cross-Cutting Issues

Two dimensions appeared as cross-cutting that is to say that they appeared in roughly equal proportion across the three main themes. These were broad characteristics of the territory, which could not be coded as related solely (or primarily) to building place, community or health. First, a certain number of unfavourable factors were mentioned, such as the difficulty to mobilise people and even more to maintain this mobilisation for any length of time. Other difficulties are material. However the interesting point is they were all overcome or were judged as being surmountable at the local level, which indicates a local ability to carry out projects of this nature.

The second cross-cutting dimension is that of the political and ideological choices that are played out at levels other than the local one. The injunctions of neoliberal obedience which have affected the health and social security systems for over 20 years are an excellent example. However, there again it should be noted that these problems are experienced more as challenges than as insurmountable obstacles. Consequently, the community health project becomes a means either to fight these ideologies or to mitigate their effects on more vulnerable populations.

**Table 6.** Overview of dimensions and related main themes

Nodes	Hierarchical nodes	Explanatory dimensions	Main themes
*67*	*30*	Place and scale effects	**Building places**
*78*	*29*	Decompartmentalization	
*41*	*24*	Decentralization	
*30*	*16*	Size effects	
*18*	*12*	Horizontal set up	
*135*	*37*	History, Identity	**Building communities**
*51*	*21*	Empowerment, appropriation, consideration for popular knowledges	
*40*	*18*	Social context, collective challenges to overcome	
*32*	*18*	Activism, mobilization	
*18*	*13*	Resistance (to privatization, gentrification, institutionalizing	
*100*	*32*	Organization of professional practices	**Building health care**
*61*	*30*	Professional networks	
*48*	*25*	Human relations (recognition and mutual trust)	
*41*	*25*	Acknowledgement of the person as a whole	
*24*	*15*	Response to needs, quality of services	
*31*	*20*	Autonomy to manage the resources	
*40*	*23*	Obstacles	**Cross-cutting issues**
*28*	*19*	Non-local political and ideological choices	
**883**	**407**	**18**	**3+2**

### 3.6. Areal-life test of key findings

The last step of this research was to test the results obtained on new fields, thus leaving aside the inductive approach in favour of a real-life assessment. This was a form of action research, intended to check the validity of the three factors identified as key for the local construction of a community healthcare service and to test their different dimensions.

**Figure 1.**
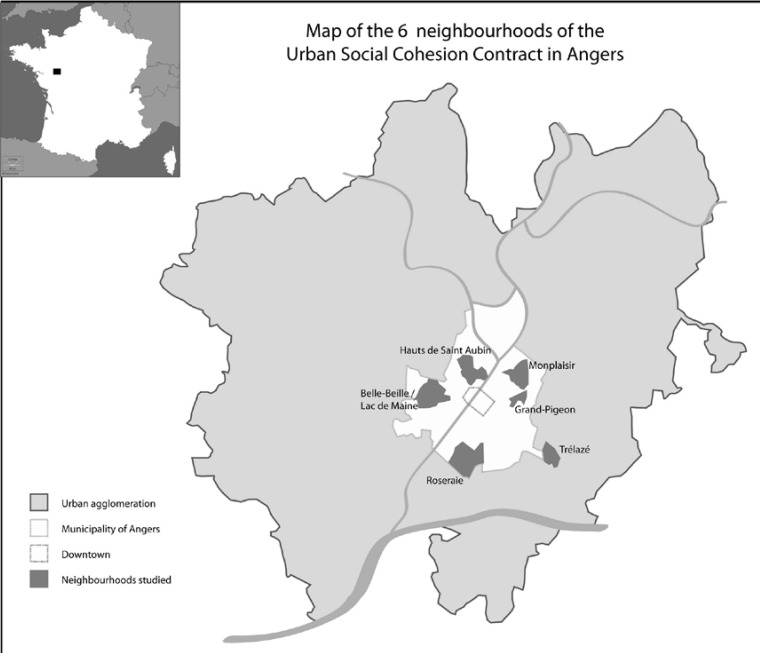
Map of the 6 neighbourhoods of the Urban Social Cohesion Contract in Angers

The setting for this test was the urban agglomeration of Angers. Six neighbourhoods were involved. They were the sites identified as priority sites within the scope of application of the Angers/Trélazé Urban Social Cohesion Contract (CUCS) ( Figure 1).

## 4. Method

A day of workshops was organised. In total, 83 actors from different territories participated, including healthcare professionals, social workers, representatives of associations, elected officials. After a plenary session presenting the results of the Montreal, Bamako and Camaçari case studies, the participants were split into 6 working groups (1 per neighbourhood). Each workshop had three sessions to work on the topics: building communities (session 1), building places (session 2) and building healthcare (session 3). The workshops were led by a previously trained facilitator. In each session the 18 dimensions identified in the previous part were spread out on a central table in the form of playing cards. The participants were asked to think about these themes from the perspective of their daily life experience. Each time they spoke they were invited to pick some of the cards and to “play” them, like trumps. In this way it was possible to pinpoint the dimensions which were mobilised according to the factor concerned (place, community, health), the actions that were underway or planned, and the context (neighbourhood).

### 4.1. Summary of workshops by factors

The first result of this assessment is the validation of the method. All the actors in these workshops reckoned the three factors that were instituted as lines of reflection to be perfectly relevant, and no dimension was seen as unsuited (or unable to be adapted) to the local context. Regarding the place, the main challenge is to bring the services provided as close as possible to the populations and ensure that they are spatially embedded in a way that corresponds to their lifestyle practices. Considering the fact that the contents and boundaries of territories are not always clearly defined, to approach the question from the perspective of the living environment can offer a broader scope of action, less restrictive than certain rigidly defined policies.

With regard to the issue of building communities, the key point is the transition from one or more informal communities to the institutional community. This change may occur when a spontaneous initiative gains importance and leaves its original small group. The initiative then needs people to act as a relay to support it. In other cases, this change may occur when professionals in a territory wish to focus on a particular community to develop their projects. In both cases, what is needed is time, the ability to listen, to identify what exists, and to gradually build an exchange and a group.

The inhabitants are commonly referred to when describing the ideas “building communities” and “building places”, but this is less the case of when evoking “building healthcare:. More often than not, the actions mentioned appear to come from the outside and are aimed at changing local perceptions and attitudes towards health issues. However, as one of the workshop participants pointed out, it is essential not to concentrate efforts only on changing behaviours but also to seek to create health-enabling conditions.

### 4.2. Neighbourhood Profiles

**Figure 2.**
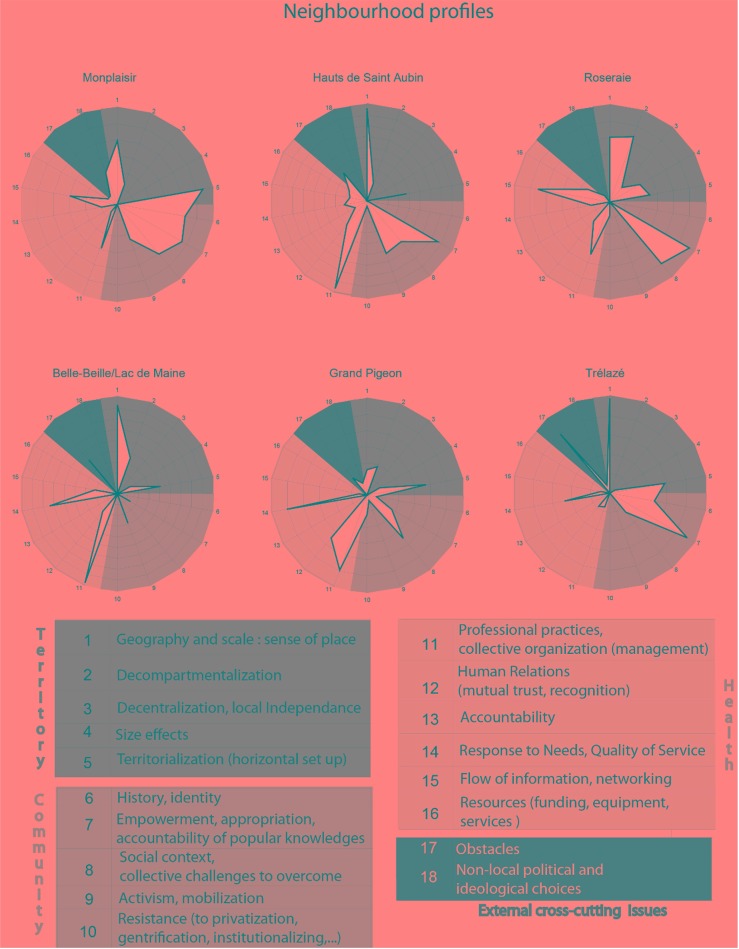
Neighbourhood profiles

The figure 2 shows, in the form of neighbourhood profiles, the three key factors of success in community health experiments and the dimensions with which they are associated.

Each dimension is represented by an axis going from the centre towards the exterior of the diagrams. The frequency with which the cards corresponding to the dimension were played during the session is graduated on the axis. This gives us a central diagram (in white on the figure) which changes shape according to the emphasis placed on any given dimension. This reveals variations between the neighbourhoods.

Generally speaking, it is clear that each neighbourhood shows a different profile, which provides an essential element to understand the transferability of local community healthcare structures. There is no single recipe and though the three factors, along with all their dimensions, are essential keys, the importance of each dimension varies according to the place. The neighbourhood profiles shown above are a tool with great potential to inform public policies about the steps to be taken in the process of creating community health services^2^.

## 5. Conclusion

This study is in phase with a growing consideration towards the determinants of health, associated with a renewal of attention for a comprehensive approach at primary level, centered on the person, also with a withdrawal of the state and an increasing devolution of the services to civil society and the community sector. Looking at the results of this study, these concerns are visible in a transverse way in the 3 main themes identified as keys for success in local health initiatives.

—building places is a means for placing determinants of health and recognizing the relevance of the local scale (and its actors) while policies, more often at a national scale, tend to emphasize the more specialized levels of care. It is in some ways a matter of putting health into place [28].

—building communities reveals the importance of understanding the spatial dimension of the societies. Place is a socially built object thus, to implement initiatives in health requires taking into account the characteristics and structures of communities. This involves more than formal boundary drawing: it is not sufficient just to trace a perimeter around a health initiative so that it “sticks” to a community's territory.

—building healthcare is a task related to the variations of scale and with the reorganizations of the health care systems to achieve more decompartmentalization and the invention of new ways for working together. Very often in that case, place constitutes a common denominator which makes it possible to take organizations out of their silo (or sets of themes) to adopt horizontal approaches, (transverse).

Finally, it is use to revisit the introduction where three questions were formulated about the factors which (i) enable community health structures to emerge, (ii) allow these initiatives to succeed and to last (ii), and explain the failures often observed when attempting to reproduce the model elsewhere or to generalise it. The results of the research presented here go a long way to answering these questions. The identification of 3 themes, 2 cross-cutting issues and the dimensions with which they are constituted provide answers to the first and second questions. The *in situ* assessment carried out in Angers, France shows that it is extremely useful to conduct a preliminary study of these factors and dimensions in the place where there is a desire to set up a structure. To do so would make it possible to avoid many pitfalls, to adapt the project to local realities and to ensure a better chance of success.

## Footnotes

1 Overlap with « building health care » point (6)

2 Overlap with the theme “building places” with the nuance that it is about delivery of services at local scale while the other batch is about considering place as a mean for enhancing cross sectorial approach.

3 Overlap with the theme “building places” with the nuance that here it is about managing the resources at a local scale while the other batch is about using place as an argument for more decentralizations.

1 When the CLSCs were merged with other public local structures to become health and social services centres (Csss)

2 Work is continuing in Angers in one of the concerned neighbourhoods with the application of these principles in the frame of a community health group and the establishment of a multidisciplinary medical centre (MSP).

## References

[1] WHO (2008). The World Health Report, 2008.

[2] Jourdan D, O'Neill M, Dupéré S (2012). Quarante ans après, où en est la santé communautaire?. San Pub.

[3] Dahlgren G (1995). European Health Policy Conference: Opportunities for the Future. Intersec Act Health.

[4] Kearns R, Moon G (2002). From medical to health geography: novelty, place and theory after a decade of change. Prog in Hum Geo.

[5] Magnussen L, Ehiri J, Jolly P (2004). Comprehensive versus selective primary health care: lessons for global health policy. Health aff.

[6] Segall M (1983). Planning and politics of resource allocation for primary health care: Promotion of meaningful national policy. Soc Sci Med.

[7] Evers A (1995). Part of the welfare mix: The third sector as an intermediate area. Voluntas: Int J Vol NonP Org.

[8] Walker DG, Teerawattananon Y, Anderson R (2010). Generalisability, Transferability, Complexity and Relevance. Ev-Bas Dec and Eco.

[9] Jolley GM, Lawless AP, Baum FE (2007). Building an evidence base for community health: a review of the quality of program evaluations. Aust Health Rev.

[10] Cambon L, Minary L, Ridde V (2012). Transferability of interventions in health education: a review. BMC Pub Health.

[11] Fournier P, Potvin L (1995). Participation communautaire et programmes de santé: les fondements du dogme. Sc. Soc. et santé.

[12] Sénécal G, Germain A, Bénard J (2002). Portrait des pratiques communautaires et locales en revitalisation urbaine et sociale sur le territoire de l'île de Montréal. rapport pour le CRDIM.

[13] Bury JA (1988). Education pour la santé; concepts, enjeux. planification.

[14] Rifkin SB, Muller F, Bichmann W (1988). Primary health care: on measuring participation. Soc sci & med.

[15] Collectif C (2006). Pointe Saint Charles: un quartier, des femmes, une histoire communautaire.

[16] Godbout JT, Martin NV (1982). Participation et innovation, in Les mobilisations populaires urbainessous la direction de Pierre Hamel, Jean-François Léonard et Robert Mayer.

[17] Desrosiers G, Gaumer B (2004). Réformes et tentatives de réformes du réseau de la santé du Québec contemporain: une histoire tourmentée. Ruptures, rev Transd en santé.

[18] Lemieux V, Bergeron P, Béguin C (2003). Le système de santé au Québec. Organisations, acteurs et enjeux.

[19] Levesque J-F, Roberge D, Pineault R, Fleurey M-J, tremblay M, Nguyen H, L. Bordeleau Le système socio-sanitaire au Québec (2007). La première ligne de soins: un témoin distant des réformes institutionnelles et hospitalières au Québec?. Gouvernance, régulation et participation.

[20] Hamel P, Jouve B (2006). Un modèle québécois?: gouvernance et participation dans la gestion publique. PU de Montréal.

[21] Brunet-Jailly J (1988). La consommation médicale des familles des personnels de l'enseignement et de la culture. compte-rendu d'une enquête réalisée en avril 1988 par les adhérents de la Mutuelle des Travailleurs de l'Éducation et de la Culture.

[22] Duponchel J-L (2004). Bilan des soins de santé primaire. Med Trop.

[23] Balique H, Ouattara O, Ag Iknane A (2001). Dix ans d'expérience des centres de santé communautaire au Mali. San publique.

[24] Medina G, Contandriopoulos A-P, Hartz Z (2005). L'intégration des soins de santé: modèle théorique d'évaluation dans un contexte de réforme du système de santé au Brésil. Bul SQÉP.

[25] Glaser BG, Strauss AL (1967-1999). Discovery of Grounded Theory: Strategies for Qualitative Research. Chicago: Aldine.

[26] Raynaud A (1984). L'intérêt de la démarche comparative en géographie. Esp Temps.

[27] Fassin D, Jeannée E, Salem G (1986). Les enjeux sociaux de la participation communautaire: les comités de santé à Pikine (Sénégal). Sci soc san.

[28] Kearns RA, Gesler WM (1998). Putting health into place: landscape, identity, and well-being. Syracuse University Press.

